# Enhanced Thermal Conductivity and Viscosity of Nanodiamond-Nickel Nanocomposite Nanofluids

**DOI:** 10.1038/srep04039

**Published:** 2014-02-10

**Authors:** L. Syam Sundar, Manoj K. Singh, E. Venkata Ramana, Budhendra Singh, José Grácio, Antonio C. M. Sousa

**Affiliations:** 1 Centre for Mechanical Technology and Automation (TEMA), Department of Mechanical Engineering, University of Aveiro, 3810-193 Aveiro, Portugal; 2 Aveiro Institute of Nanotechnology, University of Aveiro, 3810-193 Aveiro, Portugal; 3 I3N, Department of Physics, University of Aveiro, 3810-193 Aveiro, Portugal

## Abstract

We report a new type of magnetic nanofluids, which is based on a hybrid composite of nanodiamond and nickel (ND-Ni) nanoparticles. We prepared the nanoparticles by an *in-situ* method involving the dispersion of caboxylated nanodiamond (c-ND) nanoparticles in ethylene glycol (EG) followed by mixing of nickel chloride and, at the reaction temperature of 140°C, the use of sodium borohydrate as the reducing agent to form the ND-Ni nanoparticles. We performed their detailed surface and magnetic characterization by X-ray diffraction, micro-Raman, high-resolution transmission electron microscopy, and vibrating sample magnetometer. We prepared stable magnetic nanofluids by dispersing ND-Ni nanoparticles in a mixture of water and EG; we conducted measurements to determine the thermal conductivity and viscosity of the nanofluid with different nanoparticles loadings. The nanofluid for a 3.03% wt. of ND-Ni nanoparticles dispersed in water and EG exhibits a maximum thermal conductivity enhancement of 21% and 13%, respectively. For the same particle loading of 3.03% wt., the viscosity enhancement is 2-fold and 1.5-fold for water and EG nanofluids. This particular magnetic nanofluid, beyond its obvious usage in heat transfer equipment, may find potential applications in such diverse fields as optics and magnetic resonance imaging.

Working fluids, such as water (W), ethylene glycol (EG), propylene glycol (PG), engine oil, mineral oil, kerosene oil and silicon oil, are commonly used in heat transfer equipment fluids in a vast range of engineering applications. The thermal effectiveness of this equipment can be greatly enhanced by improving the thermal conductivity of the working fluids. This can be achieved by dispersing a small quantity of solid particles into the base fluids as discovered by Maxwell^1^.The augmentation of thermal conductivity of conventional fluids through the suspension of solid particles, such as millimetre or micrometre-sized particles has not been of interest for practical applications, due to problems associated with sedimentation, erosion, fouling, and increased pressure drop through the flow passages. However, dispersion of nanometer sized solid particles in base fluids, denominated as ‘nanofluids’ by Choi *et al.*^2^, has attracted much interest in the past two decades. In a seminal study, Choi *et al.*^3^ investigated the thermal conductivity of multi-walled carbon nanotubes dispersed in synthetic poly (*α*-olefin) oil nanofluids, and they found an 160% enhancement as compared to the value of the base fluid with no particles; this is a clear indication that well dispersed nanometer sized solid particles in base fluids have the potential of dramatically altering the thermal properties of the base fluid. Liu *et al.*^4^ obtained thermal conductivity enhancement of 22.4%, 12.4% 17%, 30% and 23.8% for 5% vol. of CuO-EG, 1% vol. of MWNT (multi-walled carbon nanotubes)-EG, 1.5% vol. of MWNT-water, 2% vol. of MWNT-synthetic oil and 0.1% vol. of Cu-water, respectively. Eastman *et al.*^5^ observed 40% thermal conductivity enhancement with 0.3% of Cu-EG nanofluid. Li and Peterson^6^ found thermal conductivity enhancement of 1.5-times with 6% vol. of CuO-water and 1.3-times with 10% vol. of Al_2_O_3_-water nanofluids at 34°C. Pang *et al.*^7^ obtained thermal conductivity enhancement of 10.74% and 14.29% for 0.5% vol. of Al_2_O_3_-methanol and SiO_2_-methanol, respectively, nanofluids. Hwang *et al.*^8^ obtained 11.3% thermal conductivity enhancement with 1% vol. of CNT (carbon nanotubes)-water nanofluid. Ding *et al.*^9^ also obtained an increase in the thermal conductivity of 18% for 0.1% wt. of CNT-water at 30°C. Likewise, many researchers^10^,^11^,^12^,^13^,^14^,^15^,^16^,^17^,^18^ obtained thermal conductivity enhancement for other nanofluids containing Fe_3_O_4_, SiO_2_, Ag, SiC, TiO_2_ and ZrO_2_ nanoparticles. The suitability of working fluids for heat transfer equipment depends largely on their viscosity; in addition, reliable viscosity data are required for the adequate design of the equipment in what concerns pressure loss and heat transfer. Prasher *et al.*^19^, Xiao *et al.*^20^, Chen *et al.*^21^, and Kole and Dey^22^ analyzed the effective viscosity of the nanofluids Al_2_O_3_-PG, SiC-water, TiO_2_-water, and Al_2_O_3_-car engine oil, respectively.

Nanodiamond (ND) based nanofluids are known to yield thermal conductivity enhancement; however, available information about them in the open literature is very scant. We prepared these NDs by the well-known detonation technique^23^, which leads to high thermal conductivity, good mechanical properties and chemical stability, and large surface area^24^. Recently, Ma *et al.*^24^ report the preparation of a stable nanodiamond-water nanofluid, and observed with ND 0.01% vol. a 73% thermal conductivity enhancement. Xie *et al.*^25^ obtained 18% thermal conductivity enhancement with 2% vol. of nanodiamond particles dispersed in 55% of water and 45% of EG. Torii and Yang^26^ reported pipe convective heat transfer enhancement of nanodiamond-water nanofluids. Tyler *et al*.^27^ obtained an increase of 80% in viscosity with ND 3% wt. in a nanodiamond-transformer oil nanofluid. Ghazvini *et al.*^28^ found 25% thermal conductivity enhancement with 1% wt. of nanodiamond dispersed in 20% wt. of water and 50% wt. of engine oil. Yu *et al.*^29^ measured thermal conductivity of nanodiamond-EG nanofluid and obtained 17.23% enhancement with ND 1% vol. Yeganeh *et al.*^30^ obtained 7.2% and 9.8% thermal conductivity enhancement with 3% vol. of nanodiamond-water nanofluid at 30°C and 50°C, respectively. Branson *et al.*^31^ found 12% thermal conductivity enhancement with 0.88% vol. of nanodiamond-EG nanofluid and 11% enhancement with 1.9% vol. of nanodiamond-mineral oil nanofluid, The motivation for some of the reports cited is somewhat related to the potential applications of nanodiamond in medicine and catalysis.

The use of water (W) as a working fluid in the cold regions of the world (e.g., Alaska, Canada, Northern Europe and Russia) is prevented due to its low freezing point (O°C) under normal operating conditions. Therefore, it is common practice to mix EG or PG with water to increase its freezing point. These EG-W or PG-W mixtures have been used in a variety of applications including heating industrial and residential buildings^32^. These fluids can withstand very low temperatures; however, at low temperatures (up to −50°C), EG-W mixtures have better heat transfer characteristics than the PG-W mixtures. Namburu *et al*.^34^ were the first researchers to prepare EG-W mixture based nanofluids and they considered 60% of EG and 40% of water as a base fluid for the dispersion of CuO nanoparticles. Vajjha and Das^35^ also used 60% of EG and 40% of water as a base fluid for the preparation of Al_2_O_3_ and ZnO nanofluids and measured their thermal conductivity. Naik and Sundar^36^ used 30% of PG and 70% water as a base fluid for the preparation of CuO nanofluid and verified both thermal conductivity enhancement and viscosity increase. Sundar *et al.*^37^,^38^ prepared magnetic nanofluids by dispersing Fe_3_O_4_ nanoparticles in 20:80%, 40:60% and 60:40% of EG-W mixtures for the estimation of thermal conductivity and viscosity. These nanofluids are magnetic due to the presence of the magnetic nanoparticles; magnetic nanofluids, in general, result from the dispersion of Fe_2_O_3_ (hematite), Fe_3_O_4_ (magnetite), and Ni (nickel) nanoparticles in base fluids. The saturation magnetization of Ni nanoparticles is very high compared to both hematite and magnetite nanoparticles at room temperature. Thermal conductivity and viscosity data and analyses for magnetic fluids containing Fe_3_O_4_, Fe_2_O_3_, and Ni nanoparticles are reported elsewhere^38^,^39^,^40^. The literature is scarce in what concerns synthesis and magnetic property characterization of CNT-Fe_3_O_4_, CNT-Ni magnetic nanocomposites^58^,^59^,^60^. However, it is a major challenge to prepare stable CNT-based composite magnetic fluids for engineering applications.

Considering the potential applications of magnetic nanofluids, it is desirable to produce stable magnetic nanofluids with good magnetic properties as well as high thermal conductivity. The main advantage of magnetic nanofluids over conventional nanofluids is their capability of being steered by an external magnetic field. This is an important feature in many applications, such as microfluidics and drug delivery. In the present work is reported, to the authors' best knowledge, for the first time the preparation of stable magnetic fluids based on nanocomposite of nanodiamond and nickel (ND-Ni). These nanocomposite- based magnetic nanofluids combine the good magnetic properties of nickel and the high thermal conductivity of nanodiamond. In order to produce theses magnetic nanofluids, we prepared the nanodiamond-nickel nanocomposite using the *in-situ* method and we characterized by using different techniques. We prepared the stable nanofluids by dispersing the synthesized ND-Ni nanocomposite in water, EG, and EG-water mixture for the proportions of 20:80%, 40:60%, 60:40%, respectively. We performed for these nanofluids a detailed experimental program to analyse their thermal conductivity and viscosity; as already mentioned these properties are critical in the design of heat transfer equipment.

## Results

**Figure 1:**
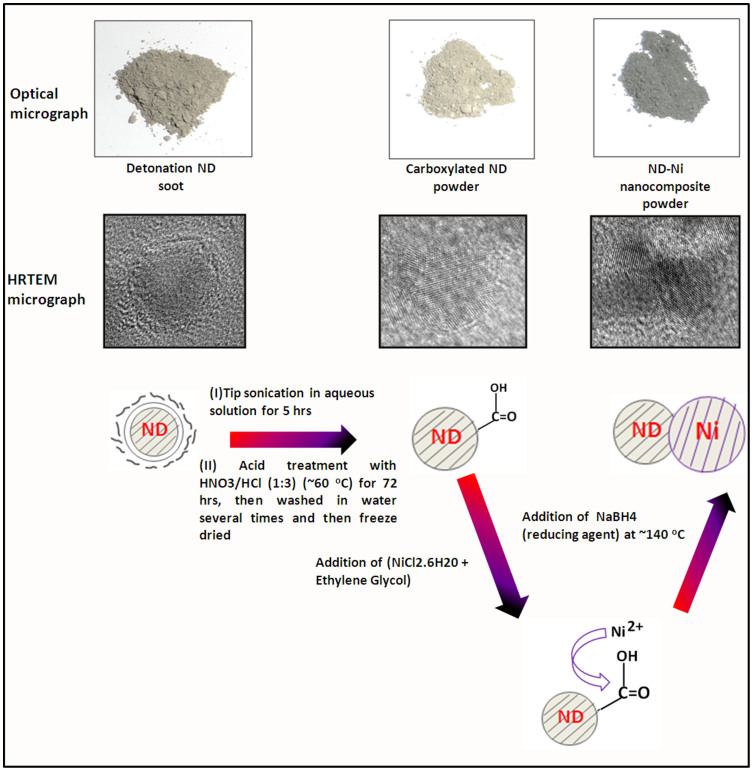
Schematic representation of in-situ growth of ND-Ni nanocomposite. (a) As received detonated nanodiamond soot, (b) c-ND powder. (c) ND-Ni hybrid nanocomposite.

**Figure 2:**
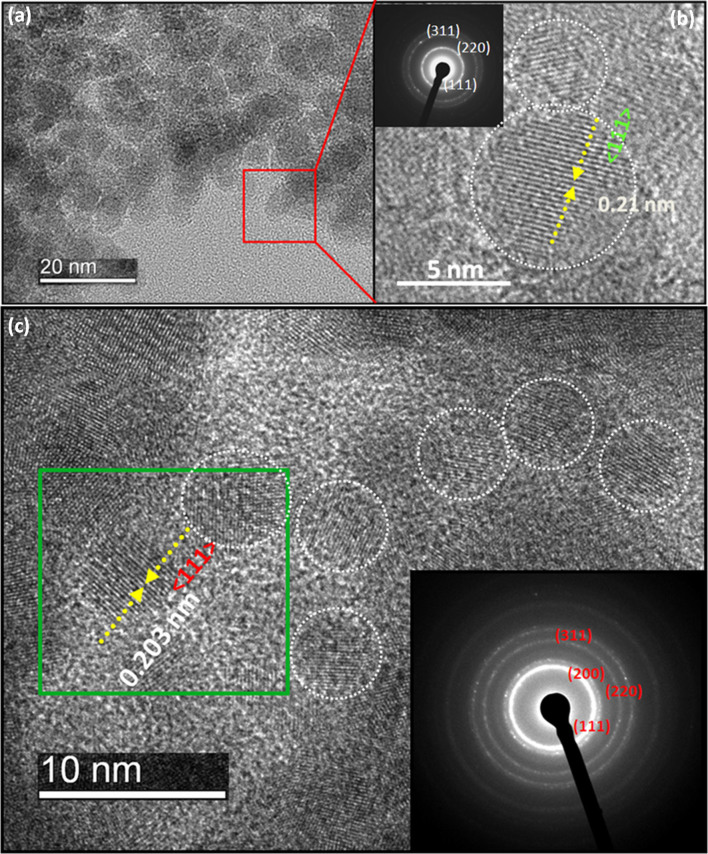
Structure of c-ND and Ni nanoparticles. (a) Bright-field TEM image of c-ND particles. (b) High-resolution TEM image of single nanodiamond particle and electron diffraction pattern (upper inset of Figure 2b) shows crystalline nature of the diamond nanoparticles. (c) High-resolution TEM image of crystalline Ni nanoparticles and corresponding electron diffraction pattern (lower inset of Figure 2c).

We present the systematic procedure and the representation of the synthesis of the ND-Ni nanocomposite in Fig. 1. As received detonated nanodiamond soot has a gray-colour appearance (Fig. 1a) due to non-diamond carbon impurities^23^. After purification and carboxylation of the detonated ND soot (Fig. 1b) presents itself like perfect nanodiamond crystals; we synthesized the crystalline ND-Ni nanocomposite using the *in-situ* technique (Fig. 1c). The subsequent transmission electron microscopy (TEM) study indicates that carboxylated nanodiamond (c-ND) nanoparticles are in the range of 4–5 nm (Fig. 2a). The high-resolution TEM (HR TEM) image (Fig. 2b) presents an interlattice plane distance of 0.21 nm, which corresponds to (111) plane of the diamond crystal. In addition, the selected area electron diffraction (SAED) pattern (upper inset in the Fig. 2b) was recorded for the c-ND particles (taken in the red square region, Fig. 2a), and it indicates the high-crystalline nature of the diamond nanoparticles. The TEM image of the synthesized Ni nanoparticles is shown in Fig. 2c, and indicates an interlattice plane distance of 0.203 nm, which corresponds to Ni (111) plane and the SAED pattern (lower inset of the Fig. 2c) taken in the green square region, further confirms the good crystalline nature of the material.

**Figure 3:**
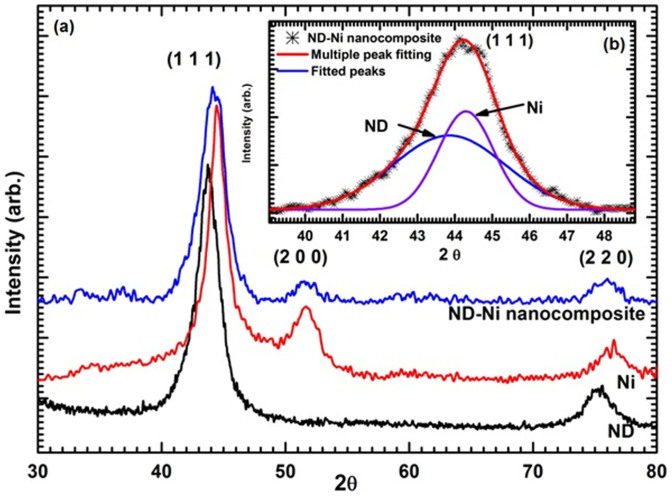
X-ray diffraction pattern of c-ND, Ni, ND-Ni nanocomposite. (a) general scan. (b) distribution of peaks fitted with multiple Gaussian functions.

We performed the crystallinity determination of the bulk ND-Ni nanocomposite by powder x-ray diffraction (XRD) and the patterns are shown in Fig. 3a. The insert (Fig. 3b) represents the distribution of peaks at maximum intensity. The peak positions of ND and Ni are very close to each other, which makes difficult to identify the exact 2θ position for ND and Ni. However, we observed peak splitting in the specific region; hence, we fitted with the multiple Gaussian peak^41^ to the obtained XRD pattern for ND-Ni nanocomposite to get the exact 2θ position for the ND-Ni nanocomposite. From the fitted curve, we found the 2θ position for ND (JCPDS No: 00-006-0675) and Ni (JCPDS No: 00-004-0850) to be 43.78° and 44.36°, respectively; these values are very close to the reported 2θ values for the (111) plane. We obtained large broadening of the peak for the (220) plane, which is due to the multiple peaks associated with ND and Ni, respectively.

**Figure 4:**
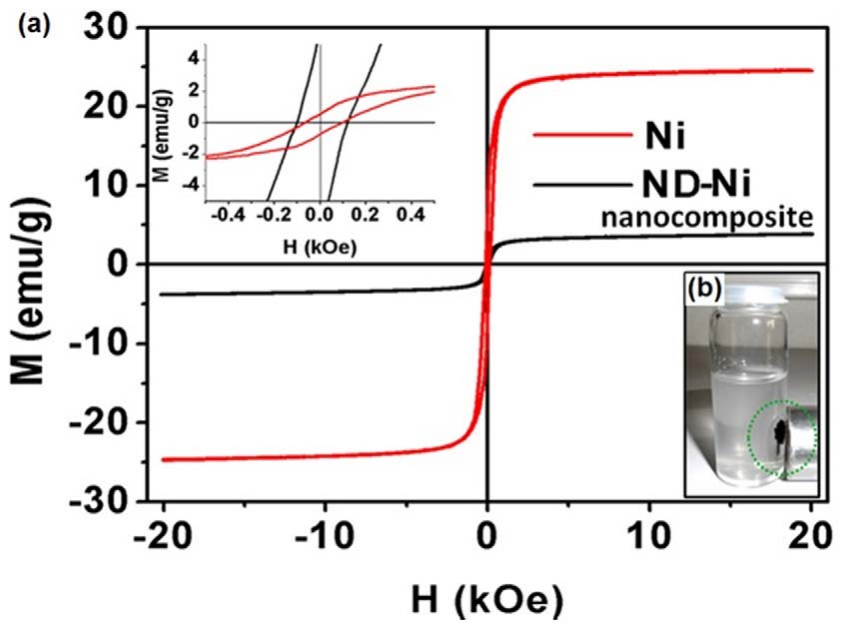
Magnetic measurement of Ni, ND-Ni nanocomposite by VSM. (a) Magnetic hysteresis loop of Ni, and ND-Ni nanocomposite. And upper inset shows coercivity (80 Oe) of pure Ni as well as ND-Ni composite samples. (b) Optical image of dispersed ND-Ni nanocomposite in water shows magnetic behaviour in the external magnetic field.

We investigated the magnetic properties of Ni nanoparticles and ND-Ni nanocomposites by measuring their magnetization-magnetic field hysteresis loops with a vibrating sample magnetometer (VSM, Cryogenic, UK). Fig. 4a shows for the Ni nanoparticles as well as for the ND-Ni nanocomposite samples ferromagnetic behaviour with saturated magnetic hysteresis loops with a coercivity of 80 Oe for both samples. The saturation magnetization for Ni is 25 emu/g, which is comparable to previously reported values for Ni nanoparticles^42^ with a particle size of 30 nm. For ND-Ni nanocomposite, the value of saturation magnetization decreases to 3.9 emu/g; this decrease in bulk magnetization is due to the large non-magnetic ND particles in the matrix of the ND-Ni nanocomposite. In the presence of the magnetic field, the non-magnetic particles act as voids and break the magnetic circuits resulting in the reduction of bulk magnetization with the increase void concentration. The total magnetization of the ND-Ni composite based on the sum of volume concentration of individual magnetizations is 3.9 emu/g, which corresponds to a bulk value of nickel weight percentage of ~16%; this result is in consonance with values reported in the literature for assemblies of magnetic/non-magnetic particles^43^. When the ND-Ni nanocomposite is dispersed in distilled water, it still shows good magnetic properties, as demonstrated by placing a magnet near to the glass beaker (Fig. 4b).

**Figure 5:**
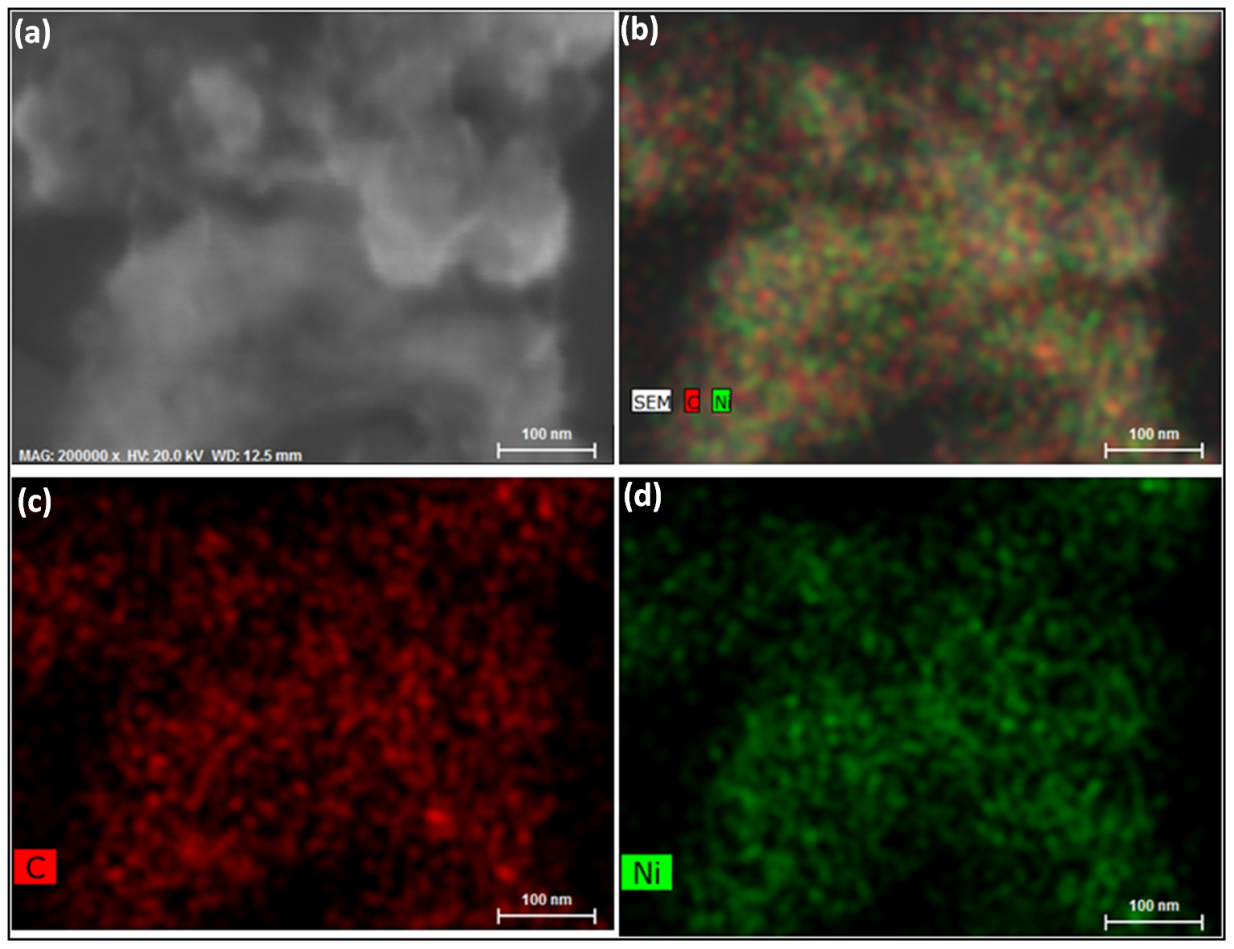
Surface morphology and elemental mapping of ND-Ni nanocomposite (a) SEM image of ND-Ni nanocomposite on copper grid and corresponding elemental mapping (b–d).

**Figure 6:**
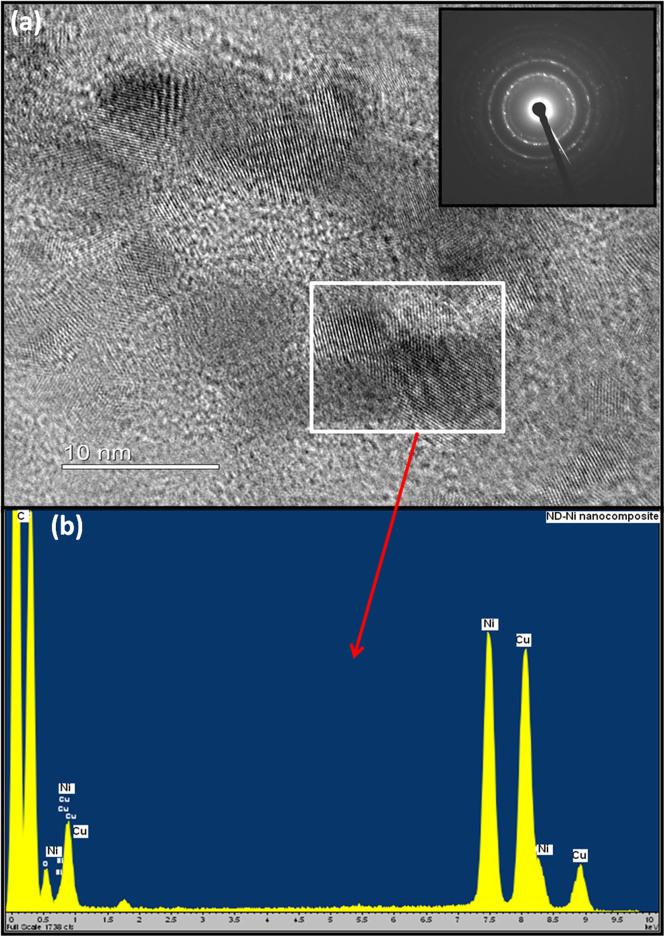
High-resolution TEM image of ND-Ni nanocomposite on copper grid (a) and electron diffraction pattern (upper inset of Figure 6a) shows crystalline in nature. (b) EDX spectra.

**Figure 7:**
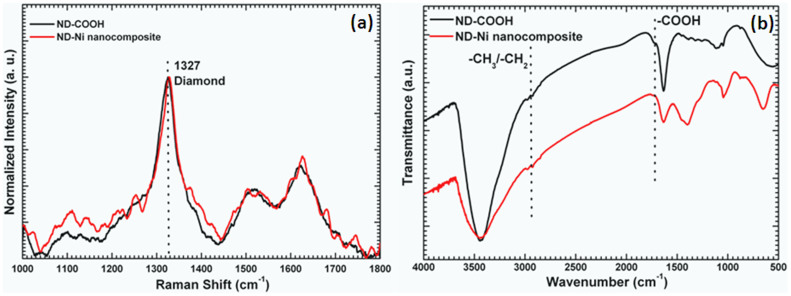
Raman spectroscopy (a) and (b) FT-IR spectrum of c-ND and ND-Ni nanocomposite.

**Figure 8:**
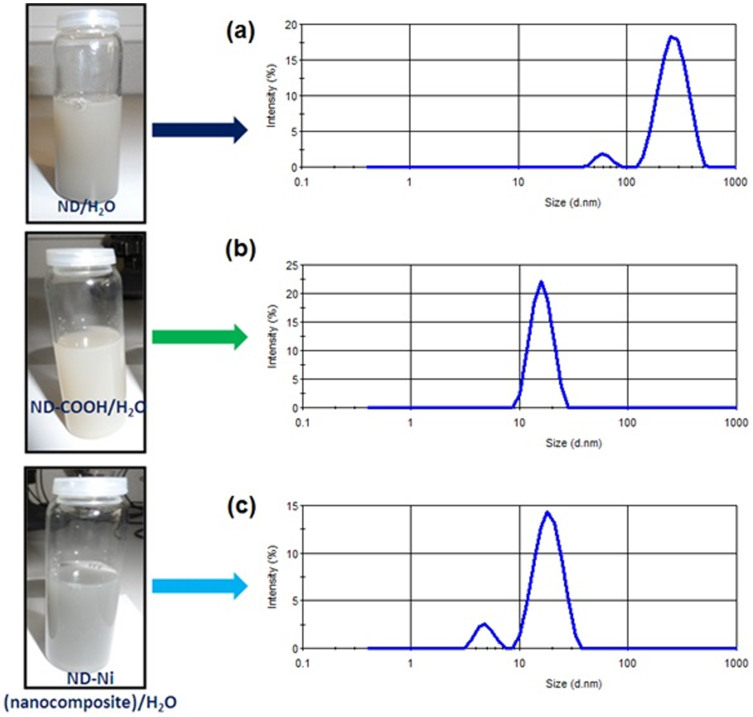
Average particle size distribution of (a) as received detonated ND soot (b) c-ND, and (c) ND-Ni nanocomposite dispersed in water.

We present the scanning electronic microscopy (SEM) of the ND-Ni nanocomposite and its corresponding elemental mapping in Fig. 5(a–d). The high-resolution TEM and energy-dispersive X-ray spectroscopy clearly shows the formation of the ND-Ni nanocomposite. In addition, the crystalline nature of this hybrid composite is confirmed by the SAED pattern (upper inset of Fig. 6) registered for the white rectangle area shown in the Fig. 6. We used Raman spectroscopy to provide information on the structure composition and homogeneity of the c-ND and ND-Ni nanocomposite. Raman spectra comparison between c-ND and ND-Ni nanocomposite is reported in Fig. 7a and it can be clearly observed the diamond peak of the ND-Ni nanocomposite at 1328 cm^−1^ with a shoulder at 1130 cm^−1^, which has its origin in smaller nanodiamond particles or smaller coherent scattering domains separated by defects in larger nanodiamond particles^44^. Another prominent feature in the Raman spectra of the ND-Ni nanocomposite is a broad peak between 1500 and 1700 cm^−1^, which is a superposition of the graphitic carbon band and O-H band^44^. Fourier transform infrared (FTIR) spectroscopy offers valuable information on the functional groups and absorbed molecules on the surface of c-ND and ND-Ni nanocomposite. The FTIR spectra of c-ND nanoparticles reveals the peak at ~1735 cm^−1^ is caused by the C = O stretch of carboxylic (COOH) (Fig. 7b), which further proves the covalent attachment of carboxylic groups on the surface of the ND particles. However, we did not observe this peak in ND-Ni nanocomposite during the *in-situ* growth, which means the carboxylic groups get reduced during the formation of Ni nanoparticles on the surface of ND. We determined the average particle size distribution of received detonated ND soot, c-ND, and ND-Ni nanocomposite dispersed in water using the Zetasizer Nano ZS (Malvern) instrument using dynamic light scattering (DLS). We observed an average particle size distribution of 270 nm for the ND soot (Fig. 8a), 14 nm for c-ND (Fig. 8b), and 25 nm for ND-Ni (Fig. 8c) nanocomposite nanofluids, respectively. We observed the following polydispersity (PDI) index values of 0.418, 0.236 and 0.374 for ND soot, c-ND and ND-Ni dispersed in water, respectively. We performed the stability analysis of the ND-Ni nanocomposite by using the zeta potential (Zetasizer Nano ZS, Malvern) and we report the data in the supplementary information (Fig. S1). In the present experiment, we measured the zeta potential for 3.03% wt. of ND-Ni dispsered in water and we obtained a value of −36.1 mV, which indicates the nanofluid has good stability; we observed no particle sedimentation for periods longer than one month, while conducting our thermal conductivity and viscosity measurements.

**Figure 9:**
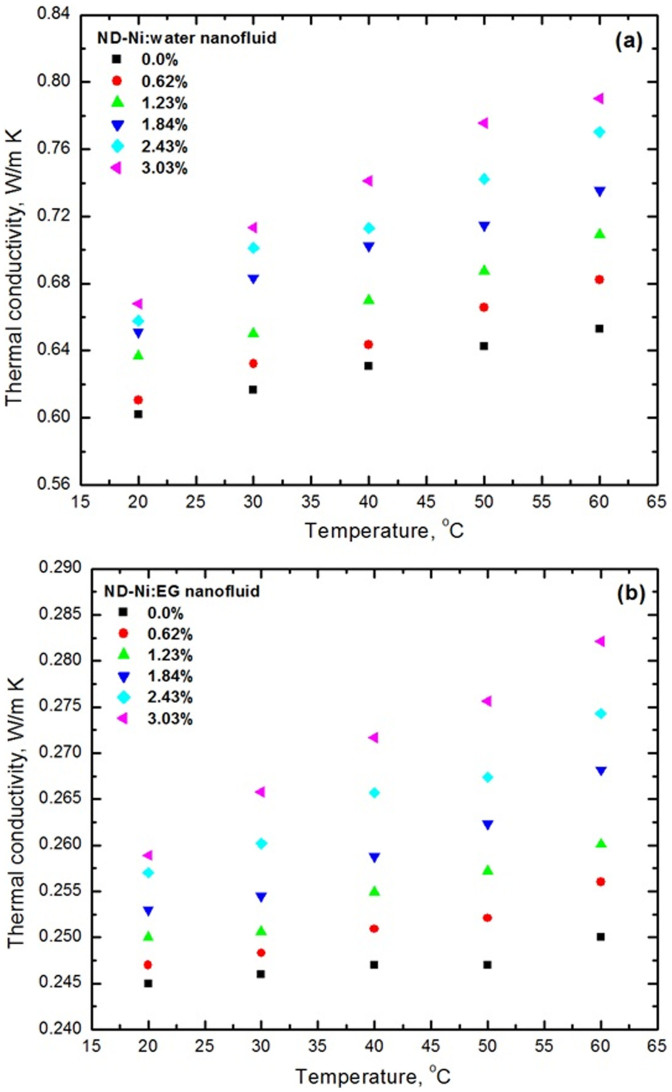
Thermal conductivity results as a function of volume concentrations and temperatures (a) ND-Ni:water (b) ND-Ni:EG.

We compared the thermal conductivity (measured by the single hot-wire probe method) of different base fluids such as water, EG, and 20:80%, 40:60% and 60:40% EG-water mixture with the AHSRAE^33^ handbook data and we observed a maximum deviation of ±2%. Measured thermal conductivity data for water and EG based ND-Ni magnetic nanofluids is presented in Fig. 9a and Fig. 9b, respectively. As expected, both water and EG based nanofluids thermal conductivity increases with increasing particle loadings and temperatures. The thermal conductivity enhancements of ND-Ni:water nanofluids for weight concentrations of 0.62%, 1.23%, 1.84%, 2.43%, 3.03% are 1.4%, 5.7%, 8.1%, 9.2%, 10.9%, respectively, at a temperature of 20°C, and 4.4%, 8.5%, 12.6%, 17.9%, 21%, respectively, at a temperature of 60°C. Similarly, thermal conductivity enhancements of ND-Ni:EG nanofluids for weight concentrations of 0.62%, 1.23%, 1.84%, 2.43%, 3.03% are 1.18%, 2%, 3.26%, 4.89%, 5.67%, respectively, at a temperature of 20°C, and 2.4%, 4.04%, 7.28%, 9.72%, 12.84%, respectively, at a temperature of 60°C. A nearly linear relationship between the thermal conductivity enhancement and volume concentration of the nanoparticles can be attributed to large regions of particle-free liquid with high thermal resistances created by highly agglomerated nanoparticles. For the case of multi-walled carbon nanotubes, TiO_2_ nano-wires, just to name a few, the relationship between thermal conductivity enhancement and volume concentration usually has a nonlinear behaviour due to high aspect ratio^45^ of these nanoparticles. However, for all cases, thermal conductivity of nanofluids increases with increasing particle concentrations and temperatures^15^,^18^,^48^.

For the particle loading of 3.03% wt., the enhancement in thermal conductivity is more pronounced for ND-Ni:water nanofluid as compared to ND-Ni:EG nanofluid by a factor of 1.65. In our experimental procedure, we prepared water and EG based ND-Ni magnetic nanofluids by the same surface modification technique and, consequently, similar covalent surface functionalization is expected. Differences in enhancement performance may be attributable to differences of the thermal boundary resistance around the nanoparticles occurring for different base fluids. In addition, the role of Brownian motion of particles in nanofluids may be an important parameter in determining the thermal conductivity enhancement and also an important factor, when the viscosity of a base fluid changes significantly with temperature, which is certainly the case for EG.

Minsta *et al.*^10^ noticed a similar trend for the thermal conductivity enhancement by for Al_2_O_3_-water nanofluid with particle sizes of 47 and 36 nm. They experimented with different particle sizes, but up to 3% vol. loading, little much variation in thermal conductivity apparent; for 9% vol. loading, the thermal conductivity of the nanofluid with 36 nm nanoparticles is higher than that of the nanofluid with 47 nm nanoparticles. This observation is a clear indication that nanoparticle size can have a significant influence upon the effective thermal conductivity of the nanofluid. It should be noted that large particles may result in their agglomeration in the base fluids, with consequent reduction in thermal conductivity enhancement. We gave careful consideration to the effective size of the nanocomposite, when preparing the nanocomposite based nanofluids; in general, for nanocomposites greater than 100 nm, it is difficult to have stable nanofluids, particularly due to particle agglomeration. In the present work, the average-size distribution of the nanocomposite is 30 nm, when dispersed in distilled water (Fig. 8c).

In addition to the characterization of the ND-Ni composite, we performed detailed measurements for the thermal conductivity of the ND-Ni magnetic nanofluid with different ratios of the EG-water mixture, its base fluid; we report the experimental results in the supplementary information (Figures S2, S3, and S4). The data may prove to be of particular interest to heat transfer equipment designers. Nanofluids prepared in various base fluids exhibit similar trend in what concerns thermal conductivity enhancement with the increase of particle loading and temperature. Under the same particle loadings of 3.03% wt. at a temperature of 60°C, the thermal conductivity enhancements for 20:80% EG-water nanofluid is 19.6%, for 40:60% EG-water nanofluid is 17.6% and for 60:40% EG-water nanofluid is 14.6%, respectively; the enhancement in thermal conductivity is clearly related to the base fluids and the presence of EG has a detrimental effect upon that enhancement. This fact may further corroborate the quasi-linearity observation of thermal conductivity enhancement with increasing loading, and the hypothesis of the thermal boundary layer resistance around each nanoparticle being a main controlling factor. The trend observed for the nanofluid is similar to that of the base fluid itself - the thermal conductivity of the EG-water mixture decreases with the increasing concentration of EG^33^. For ND-Ni particle loadings up to 1.28%, all the EG-water based nanofluids exhibit the same thermal conductivity enhancement; at larger particle loadings, the 20:80% EG-water mixture presents the highest thermal conductivity, when compared to other EG-water mixtures.

**Figure 10:**
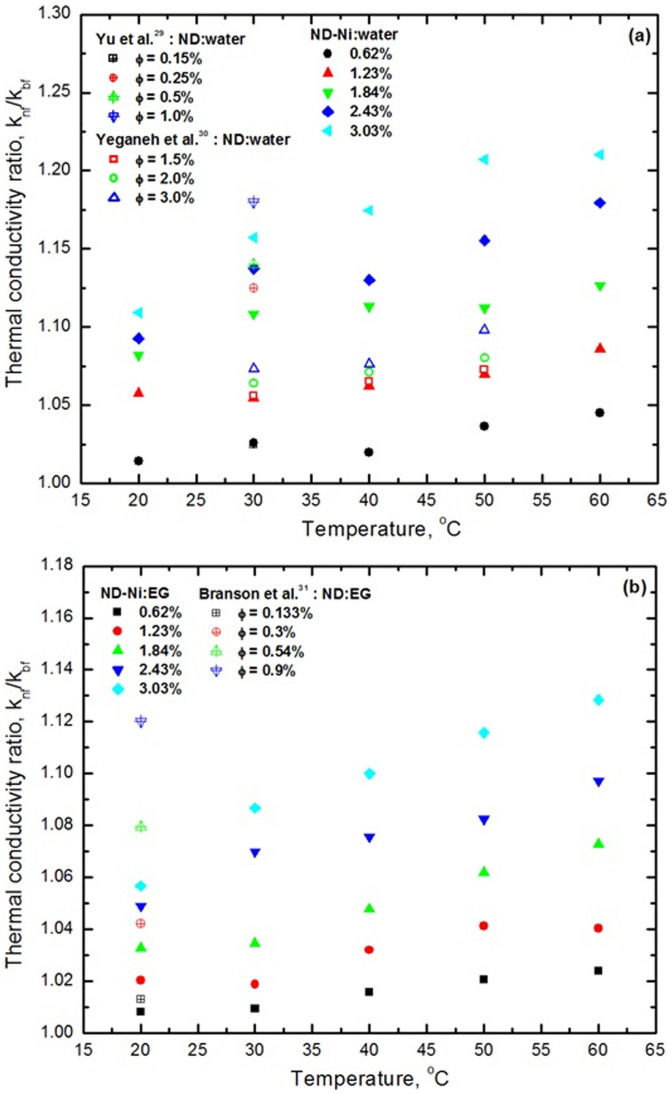
Thermal conductivity ratio as a function of volume concentrations and temperatures (a) ND-Ni:water (b) ND-Ni:EG.

The thermal conductivity ratio of ND-Ni:water and ND-Ni:EG nanofluids are reported in Fig. 10a and Fig. 10b. In the absence of literature on the ND-Ni nanocomposite based magnetic nanofluids, the present data for ND-Ni:water nanofluid is compared with the data of Yu *et al.*^29^ and Yeganeh *et al.*^30^ for nanodiamond-water nanofluid. The thermal conductivity ratio increases with increasing particle loadings and temperature; for the selected temperature measurement range, the thermal conductivity of the ND-Ni:water nanofluid has a far more pronounced enhancement than that for the nanodiamond-water nanofluid^29^,^30^ (Fig. 10a). The present data for ND-Ni:EG nanofluid is compared with the data of Branson *et al.*^31^ for nanodiamond-EG nanofluid. For comparison purpose, we prepared the EG based ND-Ni nanofluids at very low particle concentrations and at a temperature of 60°C; under these conditions, the ND-Ni:EG nanofluid presents higher thermal conductivity enhancement that that for the nanodiamond-EG nanofluids^31^ (Fig. 10b). For the same particle loading of 3.03% wt. and at 60°C, ND-Ni:water and ND-Ni:EG nanofluids present thermal conductivity enhancements of 21% and 13%, respectively. For completeness, the thermal conductivity ratio data for 20:80%, 40:60% and 60:40% EG-water based ND-Ni nanofluid are also reported in the supplementary information (Figures S5, S6, and S7). For the same particle loading of 3.03% wt. and at 60°C, ND-Ni:20:80%, ND-Ni:40:60% and ND-Ni:60:40% EG-water nanofluids have thermal conductivity enhancements of 19%, 17.6% and 15%, respectively. Once again, in the absence of information in the open literature on ND-Ni:EG-water based nanofluids, our data for the ND-Ni:60:40% EG-water nanofluids are compared with the data of Vajjha and Das^35^ for CuO nanofluid (Fig. S7); both data sets present thermal conductivity enhancement with increasing particle concentrations and temperatures. The thermal conductivity enhancement of water based nanofluids is greater than that for EG based nanofluids, and 20:80%, 40:60% and 60:40% EG-water mixture based nanofluids. For a particle loading of 3.03% wt., our observations indicate the following ranking for the thermal conductivity increase (k_w_)_nf_ > (k_20:80_)_nf_ > (k_40:60_)_nf_ > (k_60:40%_)_nf_ > (k_EG_)_nf_.

The applicability of the theoretical models of Maxwell^1^ and Hamilton-Crosser^46^ for the thermal conductivity of nanofluids may be questionable for nanocomposite based nanofluids taking into consideration the analytical formulation of these models, Eq. (1) and Eq. (2), respectively. Both models depend only on the thermal conductivity of the particles (*k_p_*), base fluid (*k_bf_*) and volume concentration (*φ*) The shortcoming is related to the uncertainty in estimating the thermal conductivity of nanocomposites, while nanoparticles such as, Al_2_O_3_, Cu, CuO, Fe_3_O_4_, SiO_2_, and TiO_2_ have their thermal conductivity well established. The theoretical models of Maxwell^1^ and Hamilton-Crosser^46^ are formulated as follows: 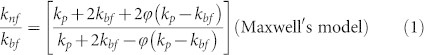

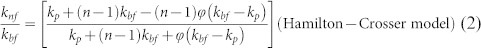
Where n is the empirical shape factor given by 3/*ψ*, and *ψ* is the particle sphericity, defined as surface area of a sphere (with the same volume as the given particle) to the surface area of the particle; for a spherical particle the value of *ψ* is equal to 3. The thermal conductivity of nanofluids, such as Al_2_O_3_, CuO, TiO_2_ and ND, tend to increase linearly with increasing volume concentration and, for this particular behaviour; different authors^47^,^10^,^31^ proposed the following correlations:

- Pak and Cho^47^ 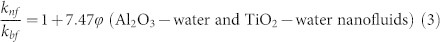
- Minsta *et al.*^10^ 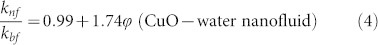

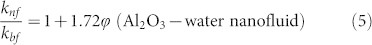
- Branson *et al.*^31^ 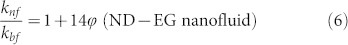

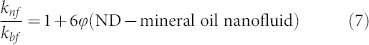
where *φ* is the percentage of volume concentration.

**Figure 11:**
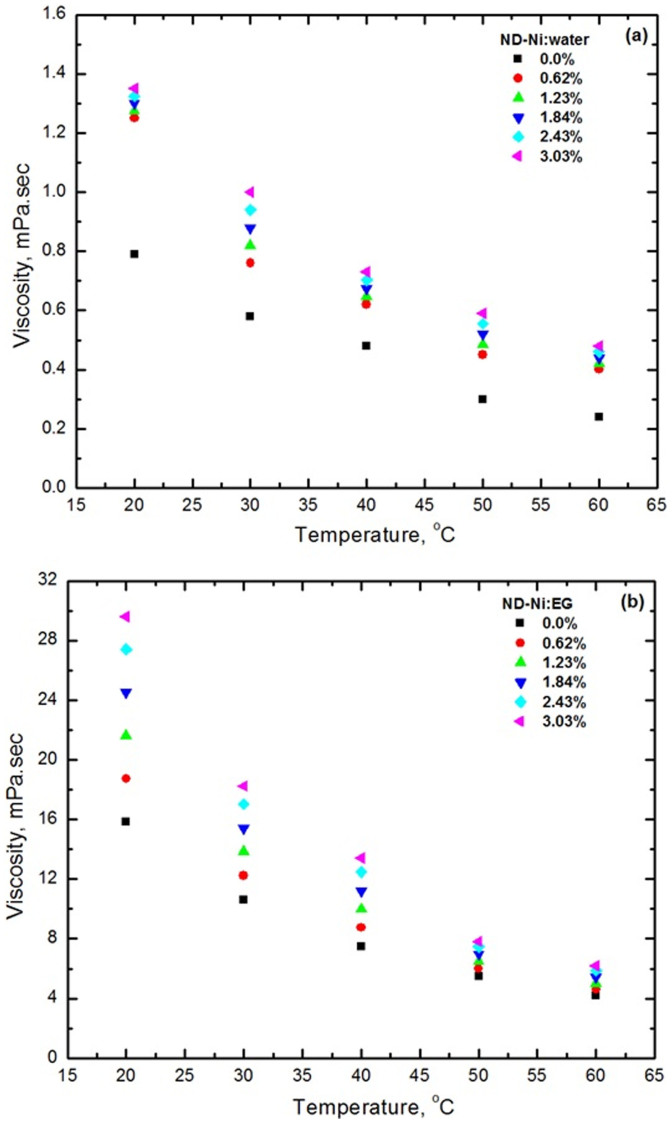
Viscosity results as a function of volume concentrations and temperatures (a) ND-Ni:water (b) ND-Ni:EG.

We employed a similar approach for our experimental thermal conductivity ratio data for the ND-Ni nanofluid; we fitted the data with a linear regression using 255 data points in a form similar to that of Eqs. (3)–(7). The validity range is for the temperature 20°C to 60°C and for the weight concentration 0.0% to 3.03%, and the resulting fitting formulation is: 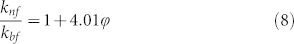
We compared our viscosity experimental data for base fluids of interest to the present study, namely water, EG, and 20:80%, 40:60% and 60:40% EG-water mixtures, with the AHSRAE^33^ handbook data and we noted a maximum deviation of ±2%. We report the experimental viscosity data for water and EG based ND-Ni magnetic nanofluids in Fig. 11a and Fig. 11b. As expected, the viscosity of both water and EG based nanofluids increases with increasing particle loading, but decreases with increasing temperature. We observed a maximum viscosity for the ND-Ni:water nanofluid with 3.03% wt. and at 60°C (Fig. 11a) is 2-times that of water, also at 60°C; the maximum viscosity for the ND-Ni:EG nanofluid with 3.03% wt. and at 60°C (Fig. 11b) is 1.5-times that of EG, also at 60°C. We noted that at higher concentrations the increase in relative viscosity, i.e., the ratio between the viscosity of the nanofluid and that of the base fluid, shows a nonlinear relationship with volume concentration. This may be due to the increasing particle-to-particle interaction for higher concentrations, which can alter the intra-molecular forces and consequently the viscosity.

We report our experimental viscosity data for the ND-Ni nanofluid with different ratios of EG-water mixture base fluid in Figures S8, S9 and S10 of the supplementary information. Under the same particle loadings of 3.03% wt. at a temperature of 60°C, the viscosity enhancements for 20:80% EG-water nanofluid is 2.4-times, for 40:60% EG-water nanofluid is 1.6-times and for 60:40% EG-water nanofluid is 1.9-times, respectively, as compared to the base fluid.

**Figure 12:**
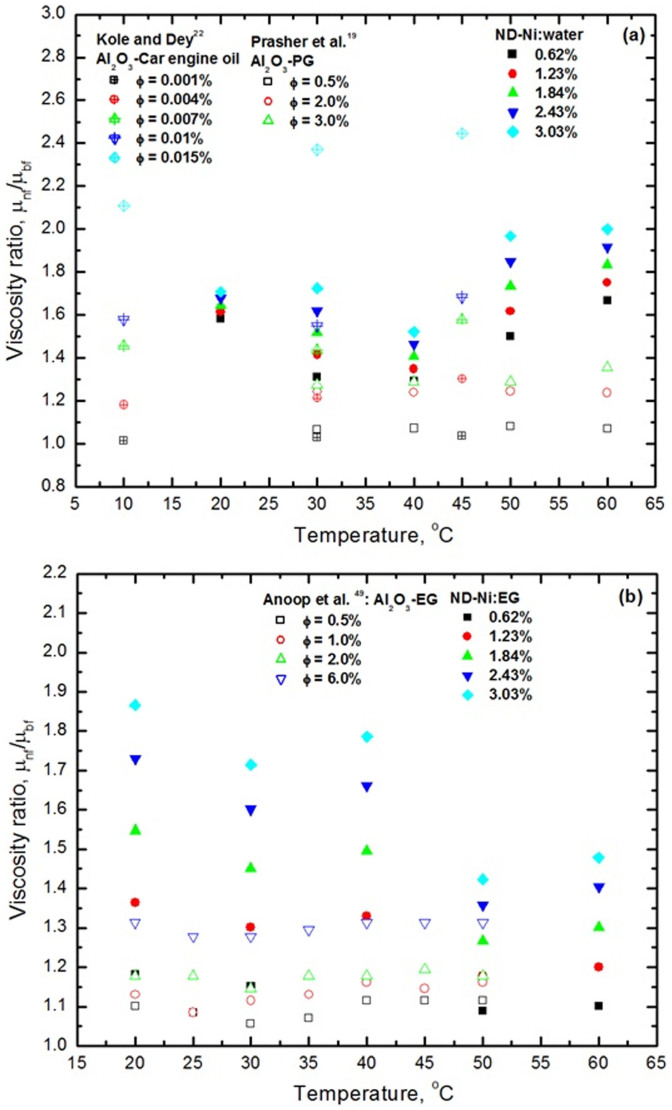
Viscosity ratio as a function of volume concentrations and temperatures (a) ND-Ni:water (b) ND-Ni:EG.

The ratio of viscosity of water- and EG- based ND-Ni nanofluids is shown in Fig. 12a and Fig. 12b. Once again, in the absence of data for ND-Ni:water nanofluid, we compare our experimental data for this nanofluid against the data of Prasher *et al.*^19^ for Al_2_O_3_-PG nanofluid and of Kole and Dey^22^ for Al_2_O_3_-car engine oil nanofluid. As already mentioned, the viscosity ratio of the nanofluid increases with increasing particle loading and temperature; for the selected temperature range of our measurements, for increasing temperature the viscosity of the ND-Ni:water does not decrease as much as that of the Al_2_O_3_-PG nanofluid^19^ (Fig. 12a). Our data for ND-Ni:EG nanofluid is compared with the data of Anoop *et al*.^49^ for Al_2_O_3_-EG nanofluid. Anoop *et al.*^49^ found the relative viscosity for all nanofluids is practically insensitive to the increase of temperature. We observed that for low particle loadings of the ND-Ni:EG nanofluid the relative viscosity is practically constant with the temperature; however, for high particle loadings, we verified that there is a small decrease of the relative viscosity with increasing temperature (Fig. 12b).

The viscosity ratio of 20:80%, 40:60% and 60:40% EG-water based ND-Ni nanofluids is reported in Figures S11, S12 and S13 of the supplementary information. We observed that, even for low particle loadings, the relative viscosity of the ND-Ni nanofluid is higher than that for the data of Sundar *et al.*^37^ for the Fe_3_O_4_ nanofluid (Fig. S11). The size of the nanocomposite appears to be an influencing parameter on the viscosity ratio. Nguyen *et al.*^50^ measured the viscosity of 36 and 47 nm of Al_2_O_3_-water nanofluids and noted that the viscosity of the 36 nm particle size is lower than that of the 47 nm particle size. The ND-Ni nanocomposite size (30 nm) (Fig. 8c) is larger than the Fe_3_O_4_ nanoparticle size (13 nm). The viscosity ratio of 40:60% EG-water based ND-Ni nanofluids decreases with increasing particle loading and temperature, whereas Sundar *et al.*^37^ observed a practically negligible variation (Fig. S12). For 60:40% EG-water based ND-Ni nanofluids, the viscosity remains unchanged with increasing temperature; however, Namburu *et al*.^34^ observed a decrease in relative viscosity with increasing temperature and Sundar *et al.*^37^ obtained an increase in relative viscosity with increasing temperature (Fig. S13). For a particle loading of 3.03% wt., our observations indicate the following ranking for the viscosity increase: (*μ*_20:80%_)_nf_ > (*μ*_w_)_nf_ > (*μ*_60:40%_)_nf_ > (*μ*_40:60%_)_nf_ > (*μ*_EG_)_nf_.

Einstein model^51^ is a well-known theoretical model to predict the effective viscosity of the solid-fluid mixture. However, the applicability of this model is limited to low volume concentrations (φ < 0.02%); the formulation of this model is given as: 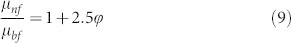
The extended Einstein's formula for use with moderate particle concentrations proposed by Brinkman^52^ is formulated as follows: 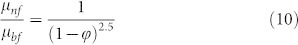
For a particle loading of 3.03% wt., the Einstein and Brinkman models predict the same value. The Einstein model for nanofluids is based on the assumption of a Newtonian fluid containing suspensions of spherical nanoparticles; it does not take into consideration temperature dependence and, in general, viscosity of liquids is strongly dependent on the temperature. Namburu *et al.*^34^,^53^,^54^ correlated viscosity of CuO, Al_2_O_3_ and SiO_2_ nanofluids with a 60:40% EG-water mixture as base fluid using an exponential form to formulate the temperature effect, namely: 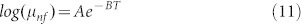

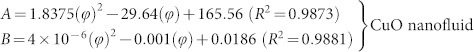

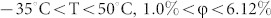

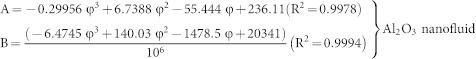

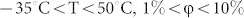

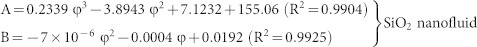

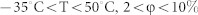
Nguyen *et al.*^50^ developed a viscosity correlation for Al_2_O_3_ (36 and 47 nm)-water and CuO (29 nm)-water nanofluids using an exponential and linear formulation, respectively, as follows: 
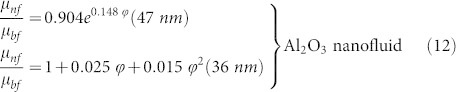

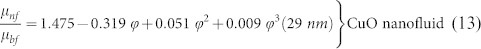

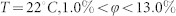
Our experimental viscosity ratio of ND-Ni nanofluids is fitted exponentially by considering 255 data points in a form similar to that of Eq. (12), and it is valid in the temperature range of 20°C to 60°C and in the weight concentration range of 0.0% to 3.03%; the formulation is expressed as: 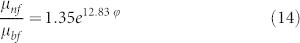


## Discussion

We prepared the magnetic nanofluids by dispersing the ND-Ni nanocomposite in water, EG, and 20:80%, 40:60% and 60:40% EG-water mixtures; we determined experimentally the thermal conductivity and viscosity for a range particle loadings and temperatures, respectively. We synthesized the magnetic ND-Ni nanocomposite by an *in-situ* growth method and characterized by using different experimental techniques. The saturation magnetization of as-synthesized ND-Ni nanocomposite is about 3.9 emu/g corresponding to a bulk value of nickel weight percentage of approximately 16%.

The maximum enhancement of the thermal conductivity of ND-Ni nanofluid for 3.03% wt. and temperature of 60°C is 21%, 13%, 20%, 15% and 18% when the base fluid is water, EG, 20:80% EG-water, 40:60% EG-water and 60:40% EG-water, respectively. Moreover, a maximum viscosity increase for 3.03% wt. of ND-Ni nanofluid prepared in water 2-times, EG 1.5-times, 20:80% EG-water 2.4-times, 40:60% EG-water 1.6-times and 60:40% EG-water 1.9-times at a temperature of 60°C compared to its base fluids, respectively.

At similar ND-Ni loadings, our performance comparison for all the base fluids used in the present study indicates that water based nanofluids have the greatest thermal conductivity enhancement. For all the ND-Ni nanofluids tested, for the same particle loading and temperature, the viscosity increase, in percentage, is greater than the thermal conductivity enhancement, also in percentage.

In what concerns the synthesis technique successfully used in the present study, also it can be applied in the preparation of various nanodiamond based magnetic nanofluids, such as ND-Fe_3_O_4_, ND-Fe_2_O_3_ and ND-Co, which may prove to be of interest for particular applications.

## Methods

### General methods

We purchased the chemicals such as ethylene glycol (EG), hydrochloric acid (HCL), nitric acid (HNO_3_), nickel chloride (NiCl_2_.6H_2_O), sodium borohydrate (NaBH_4_) and sodium chloride (NaCl) from Sigma-Aldrich chemicals, USA and used as received. We purchased the detonated nanodiamond soots from ITC (www.itc-inc.org/nanodiamond.html) with purity (>98%; cubic phase; 4–5 nm particle size).

### Disaggregation and surface modification of nanodiamond particles

As received commercial nanodiamond soot aggregates result in too large particles of the order of micron size; therefore, it is necessary to disaggregate and purify these particles in order to make nearly single nanodiamonds to make possible their use in nanofluid applications. For the purpose of disaggregation, we dissolve the nanodiamond soot in aqueous NaCl solution followed by tip sonication for 5 hours. Afterwards, we washed the solution with distilled water several times and then we froze it dried. In order to functionalize and remove the nondiamond-carbon impurities, we treated the disaggregated nanodiamond soot with a strong acidic medium (1:3 molar ratios of hydrochloric acid and nitric acid)^27^ over 72 hours and stirred under magnetic stirring at the temperature of 60°C; subsequently, we washed the particles several times with acetone and distilled water and dried in an oven at a temperature of 80°C for 24 hours. This method has the benefit of removing the nondiamond-carbon impurities and of facilitating the formation of carboxyl groups on the surface of the nanodiamond particle.

### Synthesis of nanodiamond-nickel (ND-Ni) nanocomposite

We prepared the nanodiamond-nickel nanocomposite by using the *in-situ* method. This method resorts to: (i) magnetic stirring for one hour of 20 ml of EG mixed with 0.13 g of caboxylated nanodiamond particles, then (ii) addition of 0.25 g of nickel chloride to the solution followed by further stirring of solution. When the reaction temperature of the solution reaches 140°C, we added 0.1 g of sodium borohydrate gradually and observed the formation of a black colouration, we cooled the solution down to room temperature with constant stirring. We washed the obtained nanocomposite several times with acetone and distilled water and dried in an oven at 80°C for 24 hours. We used the same procedure to synthesize the nickel nanoparticles without adding the caboxylated nanodiamond in EG for comparison purpose. We characterized the ND-Ni nanocomposite by using SEM (Hitachi; SU-70), HR TEM (JEOL 2200F TEM/STEM; 200 kV), and micro-Raman (Jobin-Yvon LabRam; 514 nm argon ion laser). We recorded the FTIR spectra with a Bruker Equinox V70 FTIR spectrometer in dry KBr pullet in the range of 400–4000 cm^−1^, and we analysed the saturation magnetization of the composite by using a vibrating sample magnetometer (VSM), Cryogenic, UK. We repeated the above procedure for the synthesis of bulk ND-Ni nanocomposite.

### Preparation of ND-Ni nanocomposite based nanofluids

We prepared the ND-Ni nanocomposite based magnetic nanofluids by dispersing into five different base fluids, namely: water, EG, and mass ratios of 20:80%, 40:60%, 60:40% of EG-water mixtures. We studied the thermal conductivity and viscosity of the nanofluids as a function of particle loading and temperature. We dispersed in 40 ml of base fluids the mass of 0.25, 0.5, 0.75, 1.00, 1.25 g, respectively, of dry powder of ND-Ni nanocomposite to achieve the weight concentrations of 0.62%, 1.23%, 1.84% 2.43% and 3.03%, respectively. To obtain a homogenous dispersion of ND-Ni nanocomposite in 40 ml of water, we used 0.1 ml of an aqueous dispersant NanoSperse AQ (http://www.nano-lab.com/dispersant-suspensions.html)^55^, subsequently we kept the mixture in an ultrasonication bath for one hour; then, we added 0.25 g of the ND-Ni nanocomposite to the solution, which we kept for another 90 minutes in the ultrasonic bath (Fig. 8c). The same procedure is repeated for the preparation of different weight concentrations of EG, and 20:80%, 40:60% and 60:40% EG-water based nanofluids.

### Measurement of thermal conductivity

We performed the thermal conductivity measurements with the KD-2 pro instrument (Decagon Devices, USA). We used the sensor needle KS-1, which is made of stainless steel having a length of 60 mm and a diameter of 1.3 mm. This needle closely approximates the infinite line heat source, which gives the least disturbance to the sample during measurements. The sensor needle measures the thermal conductivity with an accuracy of 5% in the range of 0.2–2 W/m K, which meets the ASTM^56^ and IEEE^57^ standards. We recorded 10 measurements in one hour for each weight concentration and temperature to ensure that the sample was at thermal equilibrium. We calibrated the instrument with the known thermal conductivity of glycerol, which has a value of 0.21 W/m K; the measured value is 0.20 W/m K. We measured the thermal conductivity of the different nanofluids for the weight concentration range of 0.62% to 3.03% and for the temperature range of 20°C to 60°C.

### Measurement of viscosity

We used the A & D vibro-viscometer (SV-10, Japan) to measure the viscosity of the different nanofluids as a function of particle concentration and temperature. The vibro-viscometer consists of a fluid filling cup, two gold coated plates and a PT-100 sensor with an accuracy of 0.1°C. The temperature of the fluid filling cup is controlled with a Julabo (Germany) temperature controller bath. The quantity of 40 ml of each nanofluid is placed in the cup, which is laid on the table and the height is adjusted to the mark given on the vibrating plates. The viscometer measures the viscosity in the range of 0.3 mPa. s to 10,000 mPa.s with an accuracy of ±0.01 mPa.s. Each nanofluid sample is loaded one by one in the fluid filling cup, and we conducted the measurements in the temperature range of 20°C to 60°C with an interval of 10°C. We recorded all the viscosity readings, once the system reached steady state conditions. We allowed 20 minutes to stabilize the temperature of each nanofluid to be measured.

## Author Contributions

L.S.S. & M.K.S. developed the synthesis of nanodiamond-nickel nanocomposite and performed thermal conductivity and viscosity measurements, involved in writting the manuscript. B.K.S. & E.V.R. involved in XRD, Raman and VSM measurements. A.C.M.S., J.G. & M.K.S. involved in discussion and correction of the manuscript. All authors participated equally in discussions, analysis of the results and writing of the manuscript.

## Supplementary Material

Supplementary Information Supplementary information
