# Supplementary material for: Enhanced Thermal Conductivity and Viscosity of Nanodiamond-Nickel Nanocomposite Nanofluids
Source: Sci Rep. 2014 Feb 10;4:4039. doi: 10.1038/srep04039 (PMC3918687; doi:10.1038/srep04039)
Supplement: Supplementary Information [file srep04039-s1.doc]

**Supplementary Information**

**Enhanced Thermal Conductivity and Viscosity of Nanodiamond-Nickel Nanocomposite Nanofluids**

**L. Syam Sundar^1^,*, Manoj K. Singh^1,2^,*, E. Venkata Ramana^3^, B.K. Singh^1^, José Grácio^1,2^, and Antonio C.M. Sousa^1^**

^1^Centre for Mechanical Technology and Automation (TEMA), Department of Mechanical Engineering, University of Aveiro, 3810-193 Aveiro, Portugal.

^2^Aveiro Institute of Nanotechnology, University of Aveiro, 3810-193 Aveiro, Portugal.

^3^ I3N, Department of Physics, University of Aveiro, 3810-193 Aveiro, Portugal.

*Corresponding authors: sslingala@ua.pt (L. Syam Sundar), [mksingh@ua.pt](mailto:mksingh@ua.pt) (M. K. Singh)


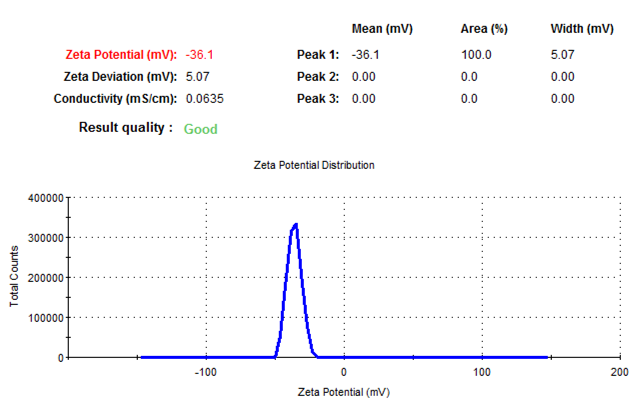


**Figure S1.** Zeta potential of 3.03 wt. % of ND-Ni nanocomposite dispersed in water.


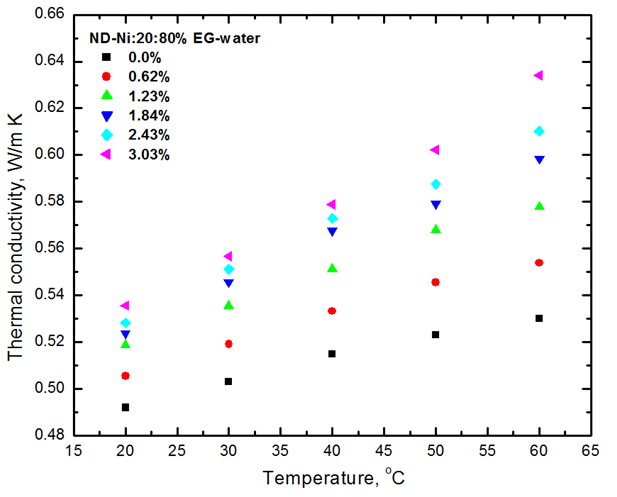


**Figure S2.** Thermal conductivity results of 20:80% EG-water ND-Ni nanofluid as a function of volume concentrations and temperatures.


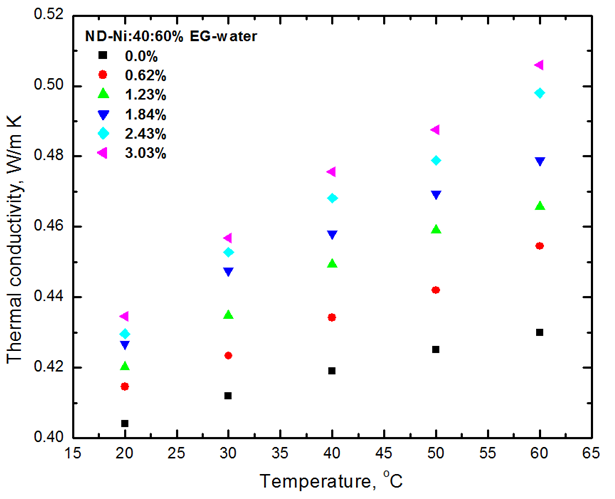


**Figure S3.** Thermal conductivity results of 40:60% EG-water ND-Ni nanofluid as a function of volume concentrations and temperatures.


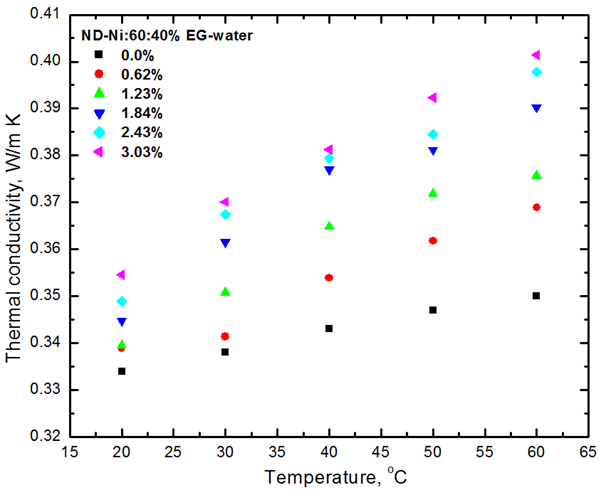


**Figure S4.** Thermal conductivity results of 60:40% EG-water ND-Ni nanofluid as a function of volume concentrations and temperatures.


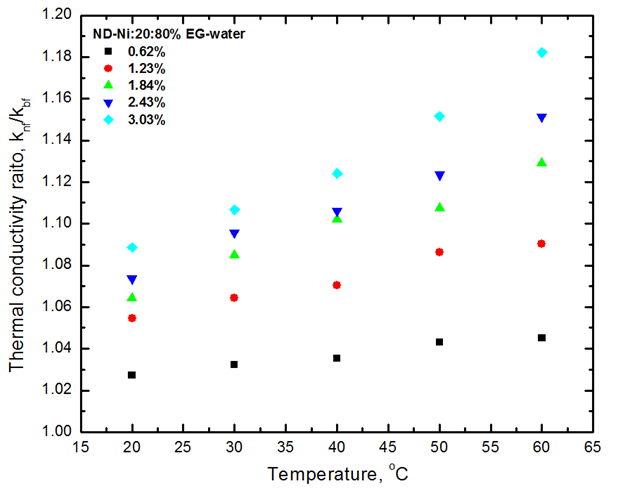


**Figure S5.** Thermal conductivity ratio of 20:80% EG-water ND-Ni nanofluid as a function of volume concentrations and temperatures.


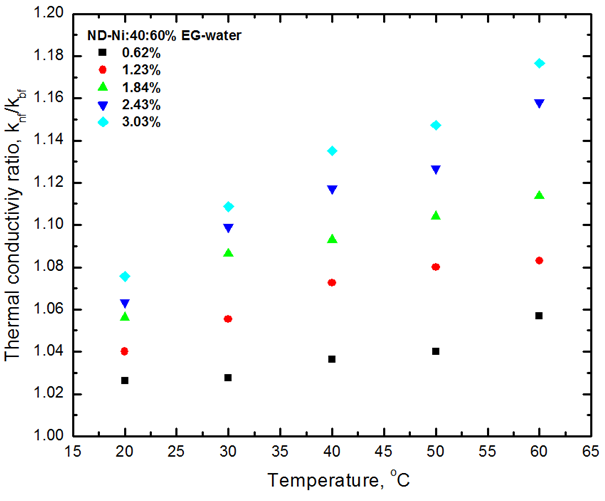


**Figure S6.** Thermal conductivity ratio of 40:60% EG-water ND-Ni nanofluid as a function of volume concentrations and temperatures.


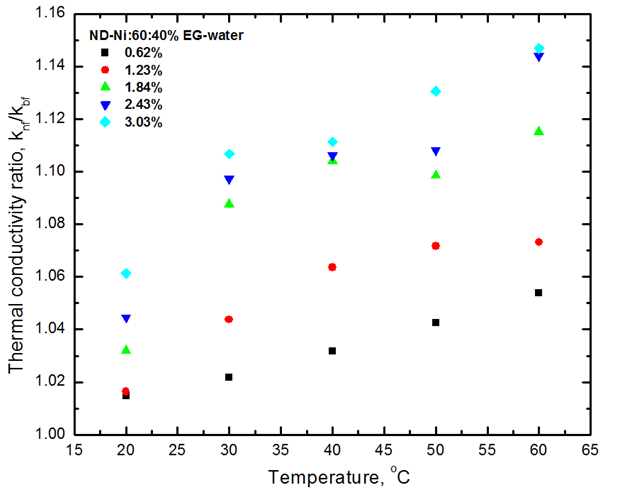


**Figure S7.** Thermal conductivity ratio of 60:40% EG-water ND-Ni nanofluid as a function of volume concentrations and temperatures.


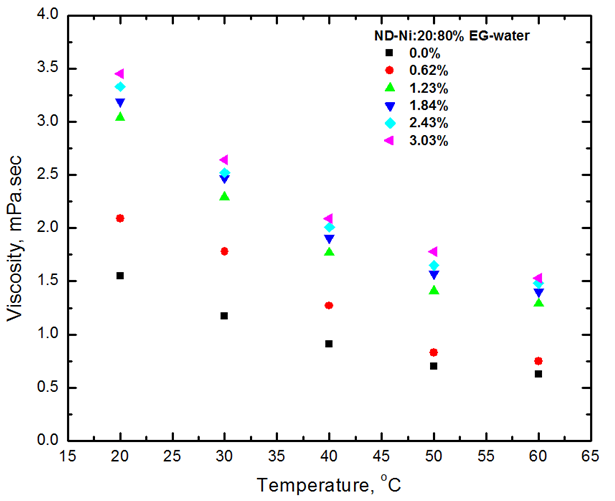


**Figure S8.** Viscosity results of 20:80% EG-water ND-Ni nanofluid as a function of volume concentrations and temperatures.


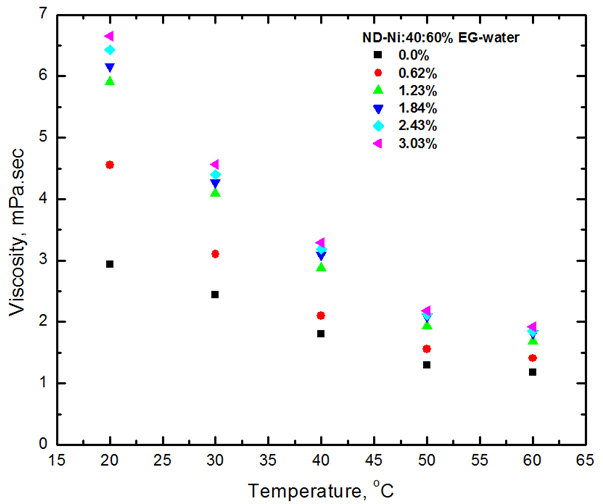


**Figure S9.** Viscosity results of 40:60% EG-water ND-Ni nanofluid as a function of volume concentrations and temperatures.


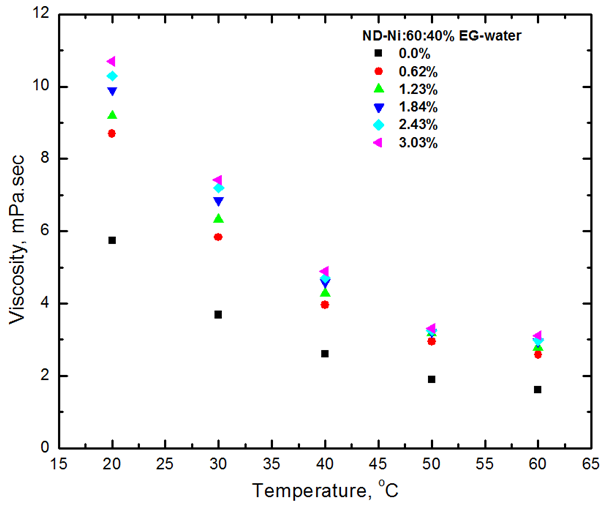


**Figure S10.** Viscosity results of 60:40% EG-water ND-Ni nanofluid as a function of volume concentrations and temperatures.


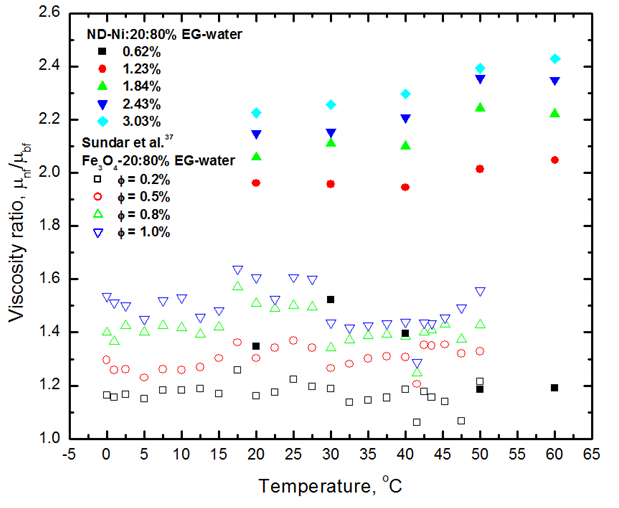


**Figure S11.** The present viscosity ratio data of 20:80% EG-water ND-Ni nanofluid is compared with the data of Sundar et al.^37^.


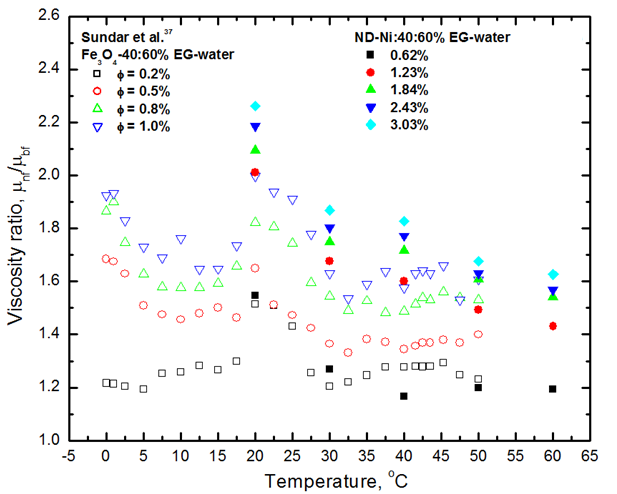


**Figure S12.** The present viscosity ratio data of 40:60% EG-water ND-Ni nanofluid is compared with the data of Sundar et al.^37^.


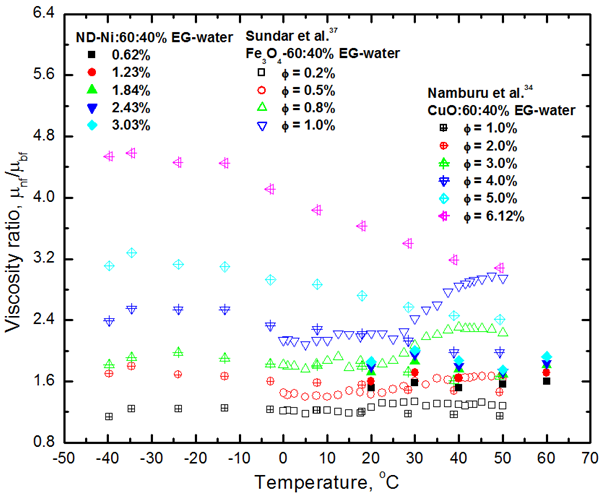


**Figure S13.** The present viscosity ratio data of 60:40% EG-water ND-Ni nanofluid is compared with the data of Sundar et al.^37^ and Namburu et al.^34^.
